# Supplementary material for: How Accurate and Precise Can We Measure the Posture and the Energy Expenditure Component of Sedentary Behaviour with One Sensor?
Source: Int J Environ Res Public Health. 2021 May 27;18(11):5782. doi: 10.3390/ijerph18115782 (PMC8198866; doi:10.3390/ijerph18115782)
Supplement: Supplementary file 1 [file ijerph-18-05782-s001.zip › ijerph-1220657-supplementary.pdf]

Supplementary Material

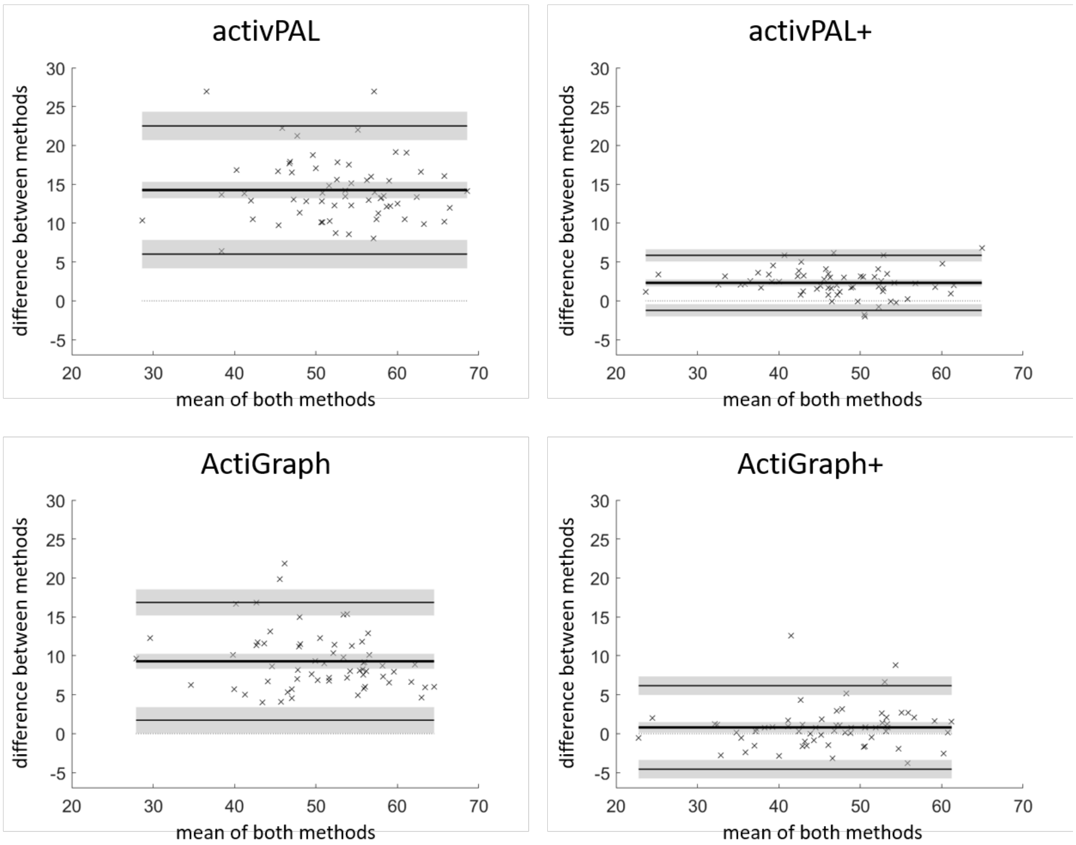

Figure S1. Bland-Altman plots for average sedentary time per day, expressed in relation to wear-time. Indicated is the bias (bold line) and the 95% limits of agreement (thin lines), both with 95% confidence interval (in grey).

Table S1. Accuracy and precision of all trained activPAL algorithms in the development and comparison sample.

	# Features	Additional Feature	Development Sample Bias	Development Sample LoA Range	Comparison Sample Bias	Comparison Sample LoA Range	Generalisability Bias	Generalisability LoA Range
Algorithms with time- and frequency-based features	1	Range (y)	3.2	13.5	5.0	10.6	1.8	-2.9
	2	Range (x)	2.9	12.8	4.2	9.4	1.3	-3.4
	3	Abs Signal Change Sum (x)	3.0	11.9	3.9	9.6	0.9	-2.3
	4	Abs Signal Change Sum (x filt)	2.8	10.4	3.5	7.7	0.7	-2.7
	5	Minimum (x angle)	2.8	8.7	3.5	7.9	0.7	-0.8
	6	Abs Signal Change Sum (VM)	2.8	9.0	3.5	7.6	0.7	-1.4
	7	Abs Signal Change Sum (y angle)	2.4	9.1	2.8	7.1	0.4	-2.0
	8	Range (x angle)	2.5	8.9	3.1	7.3	0.6	-1.6
	9	Band Power (0.3-3Hz) (x)	2.2	7.0	2.8	7.2	0.6	0.2
	10	Lag 1Hz Autocorrelation (x)	2.1	9.1	2.5	7.0	0.4	-2.1
	11	Band Power (0.3-3Hz) (y)	2.3	8.7	2.7	7.1	0.4	-1.6
	12	Lag 1Hz Autocorrelation (z filt)	0.6	1.9	2.3	7.1	1.7	5.2
	13	Range (z filt)	2.3	7.9	2.8	7.1	0.5	-0.8
	14	Coefficient of Variation (VM)	2.4	8.0	2.9	7.0	0.5	-1.0
	15	Mean Frequency (0.3-3Hz) (x)	2.4	7.8	2.9	7.1	0.5	-0.7
	16	3rd Moment (0.3-3Hz) (x)	2.3	7.6	2.8	7.1	0.5	-0.5
	17	Abs Signal Change Sum (z filt)	2.4	8.7	2.9	7.0	0.5	-1.7
		**Average (mean ± SD)**	**2.4 ±0.6**	**8.9 ±2.6**	**3.2 ±0.7**	**7.7 ±1.1**	**0.7 ±0.4**	**-1.2 ±1.9**
Algorithms with time-based features	1	Range (y)	2.5	12.9	4.1	10.1	1.6	-2.8
	2	Interquartile Range (x)	3.1	12.6	4.8	9.6	1.7	-3.0
	3	Range (x)	2.9	12.0	4.1	8.6	1.2	-3.4
	4	Abs Signal Change Sum (x)	3.1	11.4	4.1	8.9	1.0	-2.5
	5	Abs Signal Change Sum (VM)	2.6	10.7	3.2	8.7	0.6	-2.0
	6	Coefficient of Variation (x)	2.7	9.6	3.2	8.5	0.5	-1.1
	7	Standard Deviation (x)	2.3	6.5	3.0	8.3	0.7	1.8
	8	Correlation (x to z)	2.5	9.2	2.9	8.0	0.4	-1.2
	9	Standard Deviation (y)	2.8	9.4	3.3	7.7	0.5	-1.7
	10	Minimum (x)	2.1	8.6	2.6	7.8	0.5	-0.8
	11	Kurtosis (y)	2.4	8.2	2.9	7.9	0.5	-0.3
	12	5th Percentile (y)	2.0	5.5	3.1	7.9	1.1	2.4
	13	25th Percentile (x)	2.3	8.8	2.9	7.8	0.6	-1.0
	14	Skewness (x)	2.7	10.2	3.4	8.4	0.7	-1.8
	15	Correlation (x to y)	2.2	7.8	2.9	7.8	0.7	0.0
		**Average (mean ± SD)**	**2.5 ±0.3**	**9.6 ±2.1**	**3.4 ±0.6**	**8.4 ±0.7**	**0.8 ±0.4**	**-1.2 ±1.6**
Algorithms with cross-recurrent features	1	Clustering Coefficient (x)	9.6	13.1	11.0	13.2	1.4	0.1
	2	Clustering Coefficient (y)	9.7	12.9	11.0	13.0	1.3	0.1
	3	Recurrence Rate (x)	7.7	11.7	9.1	11.3	1.4	-0.4
	4	Clustering Coefficient (z)	7.9	11.3	9.1	11.2	1.2	-0.1
	5	Rec Period Density Entropy (x)	6.5	10.3	7.3	8.4	0.8	-1.9
	6	Rec Time of 2nd Type (y)	5.2	10.0	5.6	7.4	0.4	-2.6
	7	Rec Time of 2nd Type (VM)	3.8	9.6	4.4	7.7	0.6	-1.9
	8	Average Diagonal Length (x)	4.3	10.1	4.9	7.9	0.6	-2.2
	9	Clustering Coefficient (VM)	4.2	10.0	4.7	7.8	0.5	-2.2
	10	Rec Time of 2nd Type (x)	1.5	2.9	3.0	7.8	1.5	4.9
	11	Rec Period Density Entropy (z)	3.5	10.1	3.9	8.0	0.4	-2.1
	12	Average Diagonal Length (VM)	3.9	9.4	4.3	8.0	0.4	-1.4
	13	Laminarity (x)	4.1	10.5	4.7	7.8	0.6	-2.7
	14	Entropy of Diagonal Length (z)	3.6	9.3	4.1	7.8	0.5	-1.5
	15	Transitivity (x)	4.2	10.4	4.8	7.6	0.6	-2.8
	16	Trapping Time (y)	3.0	6.9	3.9	7.9	0.9	1.0
	17	Transitivity (y)	3.9	9.7	4.4	8.0	0.5	-1.7
	18	Trapping Time (x)	1.9	3.6	3.9	7.8	2.0	4.2
	19	Laminarity (VM)	3.6	8.6	4.1	7.9	0.5	-0.7
		**Average (mean ± SD)**	**4.8 ±2.3**	**9.5 ±2.6**	**5.7 ±2.5**	**8.8 ±1.9**	**0.8 ±0.5**	**-0.7 ±2.2**

Numbers presented in % of wear-time (100% equals 14.9 hours, $n_{Development} = 38$, $n_{Comparison} = 62$). The generalisability indicates the drop of the bias and range of the 95% limit of agreement (LoA Range) from the development to the comparison sample, with negative values representing an improvement. Each algorithm used one feature more as the previous algorithm, i.e. the algorithm used in the method comparison used the 12 features marked in grey. Details about the feature calculation can be found in [15] (time and frequency features) and [22] (cross-recurrent features). Abbreviations: Abs (absolute), filt (low-pass filtered data, Butterworth 2nd order, 0.5 Hz cut-off), angle (elevation angle of one axis (e.g. x for x angle) against the plane spanned by the other two axes (e.g. y and z for x angle), calculated on filtered data), VM (Vector Magnitude), SD (Standard Deviation), Rec (recurrence).

Table S2. Properties of all trained activPAL algorithms.

	# Features	Additional Feature	Method	Split Criterion	Tree Size	Max # of Splits	Min Leaf Size	Learn Rate
Algorithms with time- and frequency-based features	1	Range (y)	Bag	gdi	40	99	2′123	N/A
	2	Range (x)	Bag	deviance	14	15	4	N/A
	3	Abs Signal Change Sum (x)	Bag	gdi	13	182	81	N/A
	4	Abs Signal Change Sum (x filt)	Bag	deviance	13	222	48	N/A
	5	Minimum (x angle)	Bag	gdi	12	118′684	29	N/A
	6	Abs Signal Change Sum (VM)	Bag	deviance	47	914	3	N/A
	7	Abs Signal Change Sum (y angle)	Bag	deviance	16	38′191	87	N/A
	8	Range (x angle)	Bag	gdi	15	569	34	N/A
	9	Band Power (0.3-3Hz) (x)	Bag	gdi	123	2′723	1	N/A
	10	Lag 1Hz Autocorrelation (x)	LogitBoost	N/A	484	5	12′092	0.89
	11	Band Power (0.3-3Hz) (y)	Bag	gdi	12	49′119	64	N/A
	12	Lag 1Hz Autocorrelation (z filt)	GentleBoost	N/A	466	1′253	9	0.43
	13	Range (z filt)	Bag	deviance	10	2′472	28	N/A
	14	Coefficient of Variation (VM)	Bag	deviance	13	137′977	29	N/A
	15	Mean Frequency (0.3-3Hz) (x)	Bag	gdi	203	5′780	17	N/A
	16	3rd Moment (0.3-3Hz) (x)	Bag	gdi	29	83′429	25	N/A
	17	Abs Signal Change Sum (z filt)	Bag	gdi	11	1′243	75	N/A
Algorithms with time-based features	1	Range (y)	GentleBoost	N/A	33	25′327	2	0.00
	2	Interquartile Range (x)	LogitBoost	N/A	358	4	301	0.12
	3	Range (x)	Bag	gdi	22	7′028	154	N/A
	4	Abs Signal Change Sum (x)	Bag	gdi	469	138′156	50	N/A
	5	Abs Signal Change Sum (VM)	AdaBoost	deviance	127	8	743	0.62
	6	Coefficient of Variation (x)	Bag	deviance	11	1′363	23	N/A
	7	Standard Deviation (x)	Bag	gdi	471	15′801	6	N/A
	8	Correlation (x to z)	AdaBoost	gdi	10	84′858	189	0.06
	9	Standard Deviation (y)	Bag	gdi	18	44′860	127	N/A
	10	Minimum (x)	AdaBoost	deviance	37	923	1	0.05
	11	Kurtosis (y)	Bag	deviance	11	10′579	23	N/A
	12	5th Percentile (y)	Bag	deviance	494	68′524	3	N/A
	13	25th Percentile (x)	Bag	deviance	10	764	17	N/A
	14	Skewness (x)	Bag	deviance	11	106′758	269	N/A
	15	Correlation (x to y)	Bag	deviance	30	1′845	5	N/A
Algorithms with cross-recurrent features	1	Clustering Coefficient (x)	Bag	gdi	105	3	5	N/A
	2	Clustering Coefficient (y)	GentleBoost	N/A	356	1′858	4	0.04
	3	Recurrence Rate (x)	Bag	deviance	10	41	1	N/A
	4	Clustering Coefficient (z)	Bag	deviance	12	141	95	N/A
	5	Rec Period Density Entropy (x)	Bag	deviance	12	770	1	N/A
	6	Rec Time of 2nd Type (y)	AdaBoost	gdi	47	570	38	0.03
	7	Rec Time of 2nd Type (VM)	GentleBoost	N/A	489	56	3′550	0.02
	8	Average Diagonal Length (x)	Bag	deviance	13	438	7	N/A
	9	Clustering Coefficient (VM)	Bag	gdi	13	6′614	153	N/A
	10	Rec Time of 2nd Type (x)	GentleBoost	N/A	499	98	1	0.00
	11	Rec Period Density Entropy (z)	GentleBoost	N/A	491	2	19	0.08
	12	Average Diagonal Length (VM)	Bag	deviance	21	1′694	3	N/A
	13	Laminarity (x)	GentleBoost	N/A	498	1	12	0.00
	14	Entropy of Diagonal Length (z)	Bag	gdi	16	14′909	41	N/A
	15	Transitivity (x)	GentleBoost	N/A	303	1	371	0.00
	16	Trapping Time (y)	Bag	deviance	20	3′834	1	N/A
	17	Transitivity (y)	Bag	deviance	58	18′302	123	N/A
	18	Trapping Time (x)	Bag	deviance	99	42′565	3	N/A
	19	Laminarity (VM)	Bag	gdi	19	1′726	4	N/A

Abbreviations: Max (maximum), Min (minimum), Bag (bagging), N/A (not applicable, the different methods require different properties), Abs (absolute), filt (low-pass filtered data, Butterworth 2nd order, 0.5 Hz cut-off), angle (elevation angle of one axis (e.g. x for x angle) against the plane spanned by the other two axes (e.g. y and z for x angle), calculated on filtered data), VM (Vector Magnitude), LogitBoost (adaptive logistic regression), GentleBoost (gentle adaptive boosting), AdaBoost (adaptive boosting), Rec (recurrence).

Table S3. Average sedentary time per day measured with each single sensor method compared to POPAI, in relation to wear-time.

Method	Sedentary Time ±SD	Absolute Bias (±SE)	Relative Bias (±SE)	[95% LoA] (±SE)	RMSE	Correlation [CI]
activPAL	60.0 ± 8.1	14.3 ± 0.5	33.1 ± 2.0%	[6.0; 22.5] ± 0.9	14.8	0.87 [0.80-0.92]
activPAL+	48.0 ± 8.2	2.3 ± 0.2	5.4 ± 0.5%	[-1.2; 5.9] ± 0.4	2.9	0.98 [0.96-0.99]
ActiGraph	55.0 ± 7.5	9.3 ± 0.5	21.9 ± 1.6%	[1.7; 16.9] ± 0.8	10.0	0.89 [0.83-0.93]
ActiGraph+	46.5 ± 8.6	0.8 ± 0.3	1.9 ± 0.8%	[-4.6; 6.2] ± 0.6	2.8	0.95 [0.92-0.97]

Data given in % of wear-time, except relative bias (in % of POPAI sedentary time and thus indicating over-/underestimation) and correlation (unit less). Abbreviations: SD (Standard Deviation), SE (Standard Error), LoA (Limits of Agreement), RMSE (Root Mean Square Error), CI (Confidence Interval).

Table S4. Average sedentary time per day accumulated in bouts ≥10 and ≥30 minutes measured with each single sensor method compared to POPAI, in relation to wear-time.

Method	Sedentary Time ±SD	Absolute Bias (±SE)	Relative Bias (±SE)	[95% LoA] (±SE)	RMSE	Correlation [CI]
Sedentary time accumulated in bouts ≥10 minutes						
activPAL	49.6 ± 8.6	22.7 ± 0.9	105.5 ± 10.3%	[8.6; 36.9] ± 1.6	23.8	0.68 [0.52-0.80]
activPAL+	29.8 ± 9.5	2.9 ± 0.5	13.2 ± 2.3%	[-4.5; 10.3] ± 0.8	4.7	0.92 [0.87-0.95]
ActiGraph	32.7 ± 9.3	5.8 ± 0.4	25.0 ± 2.2%	[0.2; 11.4] ± 0.6	6.4	0.95 [0.93-0.97]
ActiGraph+	25.9 ± 9.5	-1.0 ± 0.3	-4.2 ± 1.0%	[-5.0; 3.1] ± 0.4	2.2	0.98 [0.96-0.99]
Sedentary time accumulated in bouts ≥30 minutes						
activPAL	29.2 ± 7.8	20.9 ± 0.9 #	534.7 ± 117.8%	[7.5; 34.3] ± 1.5	22.0	0.50 [0.28-0.67]
activPAL+	9.4 ± 6.0	1.1 ± 0.4	26.8 ± 7.4%	[-5.0; 7.2] ± 0.7	3.2	0.87 [0.79-0.92]
ActiGraph	11.1 ± 6.7	2.8 ± 0.2 #	49.8 ± 6.4%	[-1.1; 6.7] ± 0.4	3.5	0.95 [0.92-0.97]
ActiGraph+	8.1 ± 6.0	-0.2 ± 0.1	-2.0 ± 3.0%	[-2.4; 2.0] ± 0.2	1.1	0.98 [0.97-0.99]

Data given in % of wear-time, except relative bias (in % of POPAI sedentary time) and correlation (unit less). The hashtags mark the methods for which the bias depended on the mean of both methods. Abbreviations: SD (Standard Deviation), SE (Standard Error), LoA (Limits of Agreement), RMSE (Root Mean Square Error), CI (Confidence Interval).

Table S5. Validity of the activPAL posture classification applied to free-living data.

**activPAL Prediction**	**Ground Truth Behavior**			**Validity**		
	Sitting	Standing	Locomotion	Sensitivity	Specificity	PPV
**Contingency Table 1**						
Sitting	55.6%	0.7%	0.2%	0.95 [31]	0.98 [31]	
Standing	2.0%	25.5%	0.0%	0.88 [8]	0.97 [8]	
Locomotion	1.0%	2.9%	13.0%	0.98 [8]	0.96 [8]	
Total [16]	58.5%	29.1%	13.2%			
**Contingency Table 2**						
Sitting	58.3%	0.4%	0.0%	0.99 [32]		0.99 [32]
Standing	0.3%	25.5%	1.3%	0.90 [32]		0.94 [32]
Locomotion	0.0%	2.5%	11.7%	0.90 [32]		0.82 [32]
Total [16]	58.6%	28.4	13.0%			

Validity of the activPAL reported in Basset et al. 2014 [8] and Kim et al. 2015 [31] (Contingency Table 1), and reported in Grant et al. 2006 [32] (Contingency Table 2). Numbers applied to the sitting, standing, and locomotion classification of the activPAL in Kuster et al. 2020 [16]. Note that the numbers slightly deviate from the literature data due to different behaviors captured in each study. Since Kim et al. 2015 only reported the validity for sitting, the results of Basset et al. 2014 were added in Contingency Table 1.

Table S6. Validity of POPAI, the Posture and Physical Activity Index.

**POPAI Prediction**	**Ground Truth Behavior**					**Validity**	
	Sedentary Behavior	Active Sitting	Inactive Standing	Active Standing	Loco-motion	Sensitivity	Specificity
Sedentary Behavior	41.4%	4.0%	0.2%	0.2%	0.1%	0.925	0.919
Active Sitting	2.2%	9.2%	0.1%	0.1%	0.0%	0.678	0.973
Inactive Standing	0.4%	0.1%	10.0%	4.3%	0.3%	0.828	0.943
Active Standing	0.5%	0.1%	0.7%	10.4%	0.4%	0.630	0.979
Locomotion	0.4%	0.1%	1.1%	1.5%	12.3%	0.941	0.964
Total	44.8%	13.5%	12.1%	16.5%	13.1%		

Validity of the activPAL (average of Table S5) combined with the validity of the ActiGraph 75 counts-per-minute cut-point to separate inactive and active sitting (sensitivity of 0.951 and specificity of 0.697) and the 150 counts-per-minute cut-point to separate inactive and active standing (sensitivity of 0.932 and specificity of 0.709) reported in Kuster et al. 2021 [33].
